# A vision for a ‘microbcentric’ future

**DOI:** 10.1111/1751-7915.13262

**Published:** 2018-04-02

**Authors:** Ricardo Cavicchioli

**Affiliations:** ^1^ School of Biotechnology and Biomolecular Sciences UNSW Sydney Sydney NSW Australia

## Abstract

Microbes are the most abundant lifeforms on the planet and perform functions critical for all other life to exist. Environmental ‘omic’ technologies provide the capacity to discover the ‘what, how and why’ of indigenous species. However, in order to accurately interpret this data, sound conceptual frameworks are required. Here I argue that our understanding of microbes will advance much more effectively if we adopt a microbcentric, and not anthropocentric view of the world.

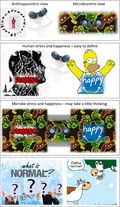

This essay is not just about ‘omics’ but it is a good positive place to start before I meander and get all philosophical. Microbiologists owe a lot to the inventors of ‘omic’ technologies and to the bioinformatic gurus and software developers who provide the means of transforming the data into biology. Thanks must particularly be paid to the scientists and technologists who paved the way for the advent of environmental omics – from dilute aquatic samples to wastewater sludge, working with environmental samples is a league beyond pure homogeneous cultures of ‘lab‐rat’ microbes. Metagenomics (Venter *et al*., 2004; García Martín *et al*., 2006) and single‐cell/virus genomics (Swan *et al*., 2011; Martinez‐Hernandez *et al*., 2017) have transcended efforts to stamp collect inventories of microbial taxa, moving it on to rationalizations of genomic blueprint potentials of individuals and populations within diverse microbial consortia. The functional ‘metaomics’, metaproteomics and metatranscriptomics have amplified understanding of microbial ecosystem processes, revealing what individual species ‘choose’ to do with their genetic potential, and how resources are allocated amongst community members (Ram *et al*., 2005; Leininger *et al*., 2006).

As bequeathed as we are with empowering omic technologies, I lament that the conceptual frameworks used for applying such technologies to environmental microorganisms have been all too often ill‐conceived. The issues appear (and are readily identifiable) in grant applications, manuscripts submitted for review, increasingly in published articles, discussed at professional society meetings, and so forth.

So, what is this dastardly problem? It relates to the notion of what is considered ‘stress’ to an environmental microorganism – what is a ‘harsh’ environment – what can be ‘tolerated’ – the whole notion of an ‘extremophile’ – in essence, a problem with having a reasonable perspective of what is ‘normal’ and therefore what might reasonably be ‘expected’ of a microorganism living in its natural environment.

All extant microbes represent a successful solution to life – they have run the evolutionary gauntlet and prevailed (at least at this snapshot in time) – which means that while we can ponder the ‘strengths’ and ‘weaknesses’ of one lifestyle versus another (e.g. lytic versus lysogenic viruses; oligotroph versus copiotroph; psychrophile versus hyperthermophile; planktonic versus biofilm), all are successful for one reason or another.

The evolutionary processes have generated way more lineages and lifestyles than are known, and in acknowledgement of this, the literature speaks of the extent of undiscovered ‘microbial dark matter’ (Rinke *et al*., 2013). As omic efforts uncover more and more lineages (Castelle and Banfield, 2018), so do discoveries (not all omics‐based) of unexpected microbial ‘lifestyles’ – nitrogen‐fixing bacteria producing methane (Zheng *et al*., 2018), Antarctic soil bacteria scavenging atmospheric hydrogen (Ji *et al*., 2017), filamentous sediment bacteria transporting electrons over centimetre distances (Pfeffer *et al*., 2012), Antarctic membrane vesicle encapsulated archaeal plasmids disseminating like viruses (Erdmann *et al*., 2017) – lots of wonderful examples. So, one wise step forward would be to resist using pigeon‐holed expectations and instead let the systems openly report back about which microbes are present, what they are doing and how they are being so extraordinary.

As to specific misconceptions, naming conventions combined with ignorance of context provides one important source of problem related to perceptions about what constitutes ‘stress’. Problems arise when concepts from one experimental system are inappropriately applied to another, often very different type of system. Consider heat shock and cold shock of the ‘lab‐rat’ *Escherichia coli*, and the respective increases of heat shock protein GroEL and cold shock protein CspA. While the overproduction of GroEL or CspA may provide signatures of a physiological stress response to a rapid, transient change in temperature for *E. coli*, it does not mean these types of proteins only fulfil roles in stress adaptation. Chaperonins (e.g. GroEL, or for that matter the ‘thermosome’) are essential protein folding machines, and some Csps, even in *E. coli*, play roles unrelated to temperature‐dependent survival. Therefore, using these types of genes or the abundance of the gene products as ‘stress markers’ to assess adaptation strategies of environmental microorganisms, could very well be uninformative or downright misleading.

It is not to say that a heat shock or cold shock could never be environmentally relevant – cold shock would be for a hyperthermophile that is ejected from its home in a hydrothermal vent into the surrounding cold water or even our favourite enteric bacterium when it is pooped out by a polar bear and finds itself stranded on the tundra. Analogous types of gross abiotic changes have relevance for other types of environmental microorganisms – for example changes in water activity, temperature and UV for microorganisms present in tidal zones.

But where conceptual frameworks come fundamentally unstuck is when natural environmental conditions are inherently considered stressful – such as cold temperatures for a polar microbe, high salt for a microbe from a hypersaline lake, high barometric pressure for a deep‐sea microbe. When this occurs, the concept of ‘tolerance’ is invoked and questions posed as to how the indigenous microorganisms tolerate such stresses. This can even lead to conclusions that the natural microbiota is not well adapted to their native environment. But the reality is that in most cases, evolution has run its course and provided a solution for competitiveness in that specific environment, and more often than not the indigenous microbes require, or are at least are ‘happy’ living under those natural environmental conditions (Cavicchioli, 2016).

Unfortunately, the advent of the term ‘extremophile’ has a lot to answer for in this regard. For all its value in highlighting the diverse environments that are capable of sustaining life, providing perspectives for the astrobiology community in searching for extraterrestrial life and capturing the imagination of the general public through to biotech companies (Cavicchioli, 2002; Cavicchioli *et al*., 2011a; Siddiqui *et al*., 2013), it has very questionable educational value. Yes, it provides a potent ‘wow’ factor, but inevitably it is linked to ‘harsh’, ‘inhospitable’, ‘stress’, ‘tolerance’ and even ‘extremotolerance’ and ‘extremotrophy’ – terms that at best, could only have meaning for an anthropocentric or perhaps *E. coli* lab‐rat‐centric view of the world. Here are just a few basic examples to illustrate why the view is problematic.

A temperature of 72°C is used for pasteurization to kill bacteria and sterilize milk; but for *Methanopyrus kandleri*, 72°C is too cold to permit growth. So, is 72°C heat or cold stress? It clearly is not an absolute and could be either, depending on the system – it also would be neither for a thermophile accustomed to growth in a hot spring at that temperature.

For salt, is 4 M NaCl stressful? Yes, if the microbe normally lives in freshwater and no if it normally lives in a hypersaline environment for which the converse (i.e. freshwater) would likely be stressful.

Is an ability to grow fast always a more competitive lifestyle than an ability to grow slow? No – marine copiotrophs can grow faster than their oligotrophic counterparts, yet oligotrophs dominate the oligotrophic reaches of the oligotrophic ocean – so the ‘rabbit’ does not always outcompete the ‘tortoise’.

Is the temperature at which fastest growth rate occurs in the laboratory (so‐called optimal growth temperature: *T*_opt_) always a good indication of adaptation to an environmental condition? No – microbes from naturally cold environments can grow faster at temperatures above their normal environmental temperatures – add kinetic energy to the system and reactions rates increase. But faster growth does not mean the microbe is ‘happy’ or functioning ‘optimally’ – it just means it is reacting to the conditions it was placed under, which just happens to speed up all of its cellular processes. Psychrophiles can even be heat stressed when growing at *T*_opt_ (Cavicchioli, 2016).

If we want to know how a microbe functions and competes effectively in its natural environment, then that is where we need to look, or at the very least, be aware of the natural conditions (biotic and abiotic factors) that support its growth (Table 1).

**Table 1:** Illustrations of differences between environmental and physiological parameters suitable for human existence versus the diversity of environmental microbial existences

Anthropocentric view of normal	Environmental microbe view of normal
Temperature: 20**°**C (room); 37°C (body)	100°C: hyperthermophile; < 5°C: ~80% of life on Earth
Oxygen	No oxygen: anaerobe
Pressure: 1 bar	1000 bar: barophile
Freshwater (consume); saline water (intracellular)	4 M NaCl: ‘extreme’ halophile; archaea high internal salt, most bacteria low internal salt
pH: neutralish	pH 2: acidophile; pH 11: alkaliphile
Food: organic (lots please)	CO_2_, minerals, NH_4_^+^: lithoautotroph; oligotroph: organic appetite (little please)
Survival: fast/strong = fit	Oligotroph: slow = fit

Through the technological capacities of omics to illuminate more and more lineages hidden within microbial dark matter, and discover the nature of interactions and functional capacities environmental microorganisms are capable of, I imagine that into the future, ‘lab‐rats’ will get increasingly sidelined and the veil affecting our perceptions will begin to lift. Enlightenment will come from attempting to interpret what we ‘see’ using the ‘eyes’ of the indigenous microbes, rather than our own (Fig. 1). Of course, microbes do not literally have eyes, but they do have other senses for responding to their environment – learning about these senses means we will in turn gain understanding about the entities using them. Comparative genomics lets us identify evolutionary pathways and physiological factors that potentially explain behaviour, and functional omic assessments enable inferences about regulatory responses to specific variables. At a broader level, we can also ‘see’ what microbes ‘see’ by observing biogeographical distinctions between communities and identifying what the environmental and dispersal forces are that explain their patterns of colonization.

**Figure 1:**
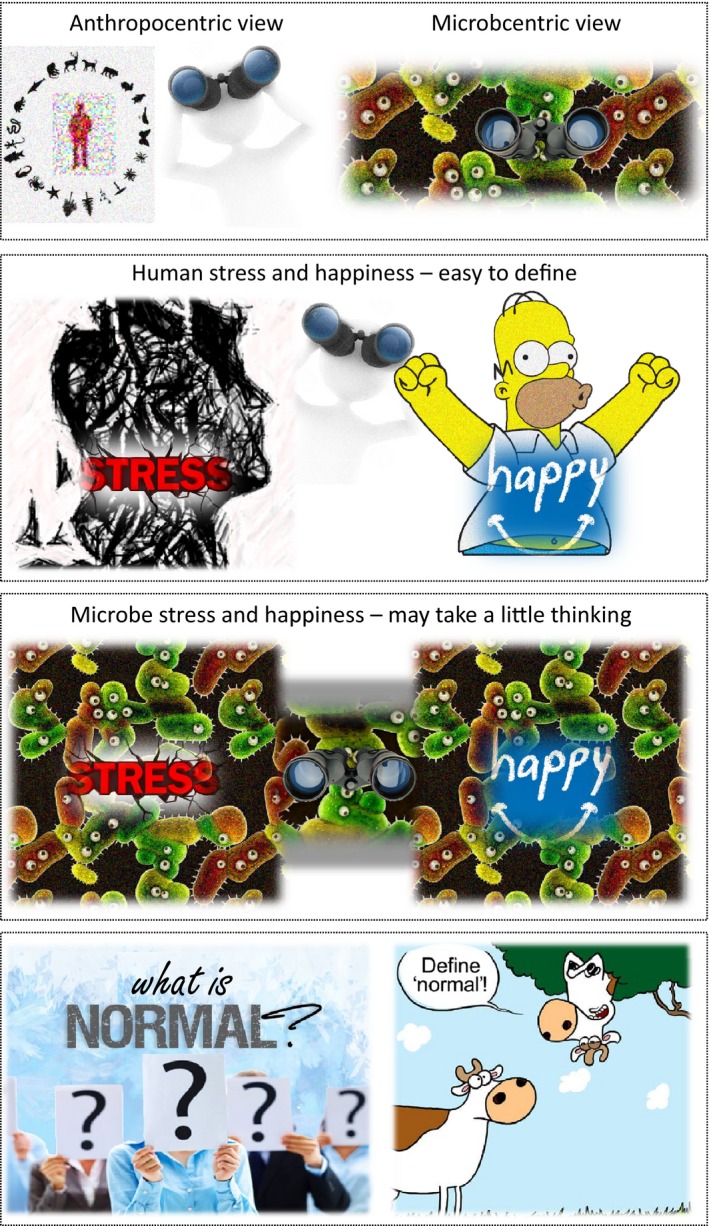
Seeing a microbcentric view of the world. The figure was constructed by modifying images obtained from Google images that linked to images distributed across numerous web sites including Filter‐Forge (https://filterforge.com).

To finish off, gaining wisdom about what normal is to environmental microorganisms (Fig. 1) will not only help pursuits of microbial adaptation/physiology, ecology and evolution, but may help provide a more rational basis for biotechnological pursuits. While it seems obvious to connect the ability of the bacterium *Thermus acquatics* to grow in hot springs with the development of its DNA polymerase for performing stable cycles of PCR, bioprospecting has rarely lived up to its apparent potential. Psychrophiles have been touted as gold mines for the discovery of cold‐active enzymes, but relatively few have been marketed (Cavicchioli *et al*., 2011b). Evolution finds workable solutions, and it finds them for organisms, not bits of organisms. If an organism is competitive in its natural environment, a successful solution has been hit upon. As long as sufficient enzyme activity exists at environmental temperatures for the cellular function of a psychrophile, the criteria for existence of the microbe have been met. But if an enzyme actually needs to sense temperature and be enzymatically active at environmental temperatures to fulfil its cellular role, that is when evolution steps up the selection pressure – an example of this is a sensor kinase from an Antarctic archaeon that has the lowest known *T*_opt_ for an enzyme from a psychrophile, and a temperature activity range that matches the growth temperature range of the organism (Najnin *et al*., 2016). Understanding the molecular mechanisms supporting the growth and lifestyle of environmental microorganisms will provide new ways of rationalizing biotechnological programs of research.

We have a lot to learn about environmental microorganisms, how they evolved into extant forms and how they do what they do – we will derive a much‐improved quality of understanding when our empowering technologies are deployed by ‘microbcentric’ operators (Fig. 1).

## References

[mbt213262-bib-0500] Castelle, C.J. , and Banfield, J.F. (2018) Major new microbial groups expand diversity and alter our understanding of the tree of life. Cell 172: 1181–1197. 29522741 10.1016/j.cell.2018.02.016

[mbt213262-bib-0001] Cavicchioli, R. (2002) Extremophiles and the search for extra‐terrestrial life. Astrobiology 2: 281–292. 12530238 10.1089/153110702762027862

[mbt213262-bib-0002] Cavicchioli, R. (2016) On the concept of a psychrophile. ISME J 10: 793–795. 26371407 10.1038/ismej.2015.160 PMC4796924

[mbt213262-bib-0003] Cavicchioli, R. , Amils, R. , Wagner, D. , and McGenity, T. (2011a) Life and applications of extremophiles. Environ Microbiol 13: 1903–1907. 22236328 10.1111/j.1462-2920.2011.02512.x

[mbt213262-bib-0004] Cavicchioli, R. , Charlton, T. , Ertan, H. , Mohd Omar, S. , Siddiqui, K.S. , and Williams, T.J. (2011b) Biotechnological uses of enzymes from psychrophiles. Microb Biotechnol 4: 449–460. 21733127 10.1111/j.1751-7915.2011.00258.x PMC3815257

[mbt213262-bib-0005] Erdmann, S. , Tschitschko, B. , Zhong, L. , Raftery, M.J. , and Cavicchioli, R. (2017) A plasmid from an Antarctic haloarchaeon uses specialized membrane vesicles to disseminate and infect plasmid‐free cells. Nat Microbiol 2: 1446–1455. 28827601 10.1038/s41564-017-0009-2

[mbt213262-bib-0006] García Martín, H. , Ivanova, N. , Kunin, V. , Warnecke, F. , Barry, K.W. , McHardy, A.C. , *et al* (2006) Metagenomic analysis of two enhanced biological phosphorus removal (EBPR) sludge communities. Nat Biotechnol 24: 1263–1269. 16998472 10.1038/nbt1247

[mbt213262-bib-0008] Ji, M. , Greening, C. , Vanwonterghem, I. , Carere, C.R. , Bay, S.K. , Steen, J.A. , *et al* (2017) Atmospheric trace gases support primary production in Antarctic desert surface soil. Nature 552: 400–403. 29211716 10.1038/nature25014

[mbt213262-bib-0009] Leininger, S. , Urich, T. , Schloter, M. , Schwark, L. , Qi, J. , Nico, I.G.W. , *et al* (2006) Archaea predominate among ammonia‐oxidizing prokaryotes in soils. Nature 442: 806–809. 16915287 10.1038/nature04983

[mbt213262-bib-0010] Martinez‐Hernandez, F. , Fornas, O. , Lluesma Gomez, M. , Bolduc, B. , de la Cruz Peña, M.J. , Martínez, J.M. , *et al* (2017) Single‐virus genomics reveals hidden cosmopolitan and abundant viruses. Nat Commun 8: 15892. 28643787 10.1038/ncomms15892 PMC5490008

[mbt213262-bib-0011] Najnin, T. , Siddiqui, K.S. , Elkaid, N. , Kornfeld, G. , Curmi, P.M.G. , and Cavicchioli, R. (2016) Characterization of a temperature‐responsive two component regulatory system from the Antarctic archaeon, *Methanococcoides burtonii* . Sci Rep 6: 24278. 27052690 10.1038/srep24278 PMC4823666

[mbt213262-bib-0012] Pfeffer, C. , Larsen, S. , Song, J. , Dong, M. , Besenbacher, F. , Meyer, R.L. , *et al* (2012) Filamentous bacteria transport electrons over centimetre distances. Nature 491: 218–221. 23103872 10.1038/nature11586

[mbt213262-bib-0013] Ram, R.J. , Verberkmoes, N.C. , Thelen, M.P. , Tyson, G.W. , Baker, B.J. , Blake, R.C. 2nd , *et al* (2005) Community proteomics of a natural microbial biofilm. Science 308: 1915–1920. 15879173

[mbt213262-bib-0014] Rinke, C. , Schwientek, P. , Sczyrba, A. , Ivanova, N.N. , Anderson, I.J. , Cheng, J.F. , *et al* (2013) Insights into the phylogeny and coding potential of microbial dark matter. Nature 499: 431–437. 23851394 10.1038/nature12352

[mbt213262-bib-0015] Siddiqui, K.S. , Williams, T.J. , Wilkins, D. , Yau, S. , Allen, M.A. , Brown, M.V. , *et al* (2013) Psychrophiles. Annu Rev Earth Planet Sci 41: 87–115.

[mbt213262-bib-0016] Swan, B.K. , Martinez‐Garcia, M. , Preston, C.M. , Sczyrba, A. , Woyke, T. , Lamy, D. , *et al* (2011) Potential for chemolithoautotrophy among ubiquitous bacteria lineages in the dark ocean. Science 333: 1296–1300. 21885783 10.1126/science.1203690

[mbt213262-bib-0017] Venter, J.C. , Remington, K. , Heidelberg, J.F. , Halpern, A.L. , Rusch, D. , Eisen, J.A. , *et al* (2004) Environmental genome shotgun sequencing of the Sargasso Sea. Science 304: 66–74. 15001713 10.1126/science.1093857

[mbt213262-bib-0018] Zheng, Y. , Harris, D.F. , Yu, Z. , Fu, Y. , Poudel, S. , Ledbetter, R.N. , *et al* (2018) A pathway for biological methane production using bacterial iron‐only nitrogenase. Nat Microbiol 3: 281–286. 29335552 10.1038/s41564-017-0091-5

